# Exogenous γ-aminobutyric Acid (GABA) Application Improved Early Growth, Net Photosynthesis, and Associated Physio-Biochemical Events in Maize

**DOI:** 10.3389/fpls.2016.00919

**Published:** 2016-06-22

**Authors:** Wu Li, Jianhua Liu, Umair Ashraf, Gaoke Li, Yuliang Li, Wenjia Lu, Lei Gao, Fuguang Han, Jianguang Hu

**Affiliations:** ^1^Crop Research Institute, Guangdong Academy of Agricultural SciencesGuangzhou, China; ^2^Key Laboratory of Crops Genetics and Improvement of Guangdong ProvinceGuangzhou, China; ^3^Department of Crop Science and Technology, College of Agriculture, South China Agricultural UniversityGuangzhou, China; ^4^Scientific Observing and Experimental Station of Crop Cultivation in South China, Ministry of AgricultureGuangzhou, China

**Keywords:** anti-oxidant enzymes, growth, maize, photosynthesis, osmoregulation

## Abstract

γ-aminobutyric acid (GABA) is an endogenous signaling molecule and involved in growth regulations and plant development, however, a little information is available on the consequences of exogenous GABA application on growth, development, and associated physio-biochemical processes in maize. The present study examined the GABA-induced regulations in early growth, net photosynthetic rate, gas exchange, osmoregulation, and enzymatic activities in three maize cultivars, i.e., Yuecainuo 6, Zhengtian 68, and Yuecainuo 2. Two levels of GABA, i.e., 0 mg L^-1^ and 50 mg L^-1^, in solution form, with total application volume of 100 ml per pot containing 15 maize seedlings were exogenously applied. Results revealed that exogenous GABA application improved seedling growth in terms of seedling length and biomass accumulation in all maize cultivars at both 3 and 7 days after treatment (DAT). It also promoted net photosynthesis and variably affected gas exchange attributes, i.e., stomatal conductance (Gs), intercellular CO_2_ concentration (Ci), and transpiration rate (Tr), as well as leaves SPAD value. Furthermore, lipid peroxidation [in terms of malondialdehyde (MDA)] under GABA treated maize seedlings were also remained variable; however, osmolyte accumulation (protein and proline) and activities of anti-oxidants enzymes, i.e., super-oxide dismutase and peroxidase were also affected differently at both 3 and 7 DAT in all maize cultivars. Furthermore, enzymes involved in nitrogen metabolism, e.g., nitrate reductase and glutamine synthetase were improved. These results suggest the involvement of GABA in various physio-metablical mechanisms which might lead to improvement in morphological growth of maize. In future, research is still needed at molecular and genetic levels to unravel the involvement of GABA-mediated regulations in growth and its associated physio-biochemical mechanisms.

## Introduction

Plants have complex mechanisms of inter and intra cellular signal transductions which play a major role in growth regulation and plant development. This system of plant signaling not only controls the growth behavior but also projects the complete life cycle or whole growth period of a plant. Plant signaling molecules are important in this regard in which they integrate external stimuli to internal plant processes for an appropriate response. Most often, plants respond to these signaling molecules at the level of their biosynthesis, transportation, and uptake or at the level when these are perceived (Ma, 2003; Bouche and Fromm, 2004; Roberts, 2007).

γ-Aminobutyric acid (GABA), four-carbon non-protein amino acid, is well recognized as an endogenous plant signaling molecule and involved in various physio-biochemical processes of a plant. For example, it triggers up the nitrate uptake and nitrate transport gene expression (*BnNrt 2*) the in *Brassica napus* and regulates 14-3-3 gene family members in seedling of *Arabidopsis thaliana* (Beuve et al., 2004; Lancien and Roberts, 2006). Development of pollen tube and its orientation is also related to GABA levels in the tobacco plants (Yu and Sun, 2007). GABA-induced alleviation of proton and aluminum stress is well explored by Song et al. (2010) at seedling stage in barley whereas regulation of gene expression associated with H_2_O_2_ and ethylene production in the roots of *Caragana intermedia* is also related with endogenous GABA application (Shi et al., 2010). Activation of antioxidant defense system to scavenge ROS and to palliate oxidative damage is also a boon of GABA in peach under chilling stress (Yang et al., 2011). Additionally, endogenous GABA levels in plants are very low; however, it was produced rapidly in plants under stressful conditions to withstand against them (Kinnersley and Turano, 2000). Exogenous application of GABA promoted morphological growth, functioning of photosynthetic machinery, gas exchange capacities, chlorophyll biosynthesis, enzymatic, and non-antioxidant responses and membrane stabilization in tomato (Luo et al., 2011). Furthermore, roles of GABA in osmoregulation, pH change, glutamate homeostasis and its action as a signaling source for nitrate uptake are important with respect to plant response to external environments (Carroll et al., 1994; Shelp et al., 1999; Masclaux-Daubresse et al., 2002; Beuve et al., 2004) whilst GABA is also involved in nitrogen metabolism (storage or transport) and C:N fluxes.

γ-aminobutyric acid is synthesized in a complicate pathway called the GABA shunt (conversion of glumate to succinate) which includes three main enzymes, i.e., glutamate decarboxylase (GAD), GABA transaminase, and succinic semialdehyde dehydrogenase (SSADH), of which GAD is the key enzyme which is responsible for irreversible α-decarboxylation of glutamate “the first step of the GABA shunt”. Secondly, GABA is catalyzed to succinate semialdehyde reversibly by the action of GABA transaminase where α-ketoglutaric acid or pyruvate acts as amino acceptors. Finally, succinic semialdehyde is irreversibly oxidized to succinate (Rhodes et al., 1999). Moreover, GABA might also be produced from γ- aminobutyraldehyde (a product of the polyamine catabolic pathway) through betaine aldehyde dehydrogenase which localized in chloroplasts and also involved in biosynthesis of glycinebetaine. Exogenous application of different plant growth regulators, phyto-hormones and growth promoters have proved their significant impacts in growth regulations in maize (Anjum et al., 2011a,b,c); however, reports on the effects of GABA application on early maize growth and its involvement in various metabolic events are very few. This study examined the GABA-induced regulations in early performance of maize seedling, photosynthetic and gas exchange capacities, and anti-oxidative defense system to protect against oxidative stress with the hypothesis that GABA may improve early performance of maize by regulating its related physio-biochemical processes.

## Materials and Methods

### Experimental Material and Growing Conditions

A pot experiment was conducted by using three popular maize varieties, i.e., Yuecainuo 6, Zhengtian 68, and Yuecainuo 2 collected from Crop Research Institute, Guangdong Academy of Agricultural Sciences, Guangzhou, China. This region has a humid subtropical monsoonal type of climate characterized by hot summers and warm winters with yearly average temperature ranged from 21 to 29°C (Li et al., 2016; Mo et al., 2016) The cultivars used in this study are well-recognized and widely grown corn cultivars in South China. Before sowing, healthy seeds with uniform color, shape and with intact seed coat were selected from the seed lot and sown in plastic pots (18 cm × 13 cm × 6 cm) containing sandy loam soil with total nitrogen 0.97 g kg^-1^, total phosphorous 0.82 g kg^-1^, total potassium 25.50 g kg^-1^, and pH 6. The pots were placed at room conditions (28° C) under sun light with light/dark period of 12/12 h and 60 ± 5% relative humidity (RH).

### Treatments

Two levels of GABA (Sigma) (0 mgL^-1^ (-GABA) and 50 mgL^-1^ (+GABA), in solution form), with total application volume of 100 ml per pot and split into twice (50 ml for 15 days after sowing (DAS), the rest 50 ml were applied at 16 DAS), were exogenously applied to the seedlings (at 2–3 leaves stage). The pH of the solution was 7.0 (the same as the control (distilled water application). For each variety, 25 seeds per pot were sown with 10 pots per treatment whilst at first fully expanded leaves, 15 seedlings with consistent growth were kept in each pot and other pants were thinned out. The treatments were arranged in completely randomized design (CRD).

### Observations

#### Photosynthesis and SPAD Value

The photosynthesis characters were measured with the portable photosynthesis system (LI-6400, LI-COR, USA) during 9:00–11:00 am according to Pan et al. (2015). The top fully expanded leaves of two represented seedlings from each three different pots of each cultivar were selected to measure the net photosynthetic rate (Pn), stomatal conductance (Gs), intercellular CO_2_ concentration (Ci), and transpiration rate (Tr) with the following adjustments: photo-synthetically active radiation at leaf surface was up to1200 μmol m^-2^ s^-1^, molar flow of air per unit leaf area was 500 μmol s^-1^, ambient CO_2_ concentration was 400 μmol mol^-1^, air temperature was 30°C, and RH was 60%. The SPAD values represented as chlorophyll contents were recorded with a SPAD meter ‘SPAD-502’ (Konica Minolta, Japan) according to Wu et al. (1998) that provided a precise, rapid and non-destructive estimation of leaf chlorophyll contents. The average SPAD values (determination was done at the upper 1/3rd, middle, and lower 1/3rd SPAD value in leaves) was considered as the relative chlorophyll content.

#### Physiological Parameters

To determine physiological parameters, a total number of 25 seedlings were harvested from 5 pots (5 seedlings per pot), separated into leaves and roots and immediately dropped into liquid nitrogen for 30 s then stored at –80° C till biochemical analyses.

The soluble protein content was measured base on the method of Bradford (1976) by using G-250. Briefly, 0.1 g of tissue was homogenized with 1 ml of 0.1 M ice-cold phosphate buffer, pH 7.0, containing 1% polyvinylpolypyrrolidone. The resulting homogenate was centrifuged at 10,000 *g* at 4° C for 10 min, and the supernatant was immediately used for soluble protein measurements. The supernatant was mixed with the comassie brilliant blue-G250 solution and the absorbance of the reaction mixture was read at 595 nm with a spectrophotometer. Protein content was determined from a standard curve (bovine serum albumin) and expressed as μg g^-1^.

The proline content was measured by method of Bates et al. (1973) by using ninhydrin. The fresh sample of 0.3 g was homogenized in 3%sulphosalycylic acid and homogenate filtered through filter paper. After addition of acid ninhydrin and glacial acetic acid, the mixture was then heated in water bath at 100°C for 1 h. Reaction was then stopped by using ice bath. After reaction the absorbance of the red chromosphere in the toluene fraction was measured at 520 nm. Proline content was determined using calibration curve and expressed as μmol g^-1^.

The malondialdehyde (MDA) content was measured by the method of Chen and Wang (2006). Fresh sample (0.3 g) was homogenized in 5 ml of 10 % trichloroacetic acid and centrifuged at 4000 *g* for 15 min. To each 2 ml of the supernatant, 2 mL of 0.6% thiobarbituric acid in 10% TCA was added. The mixtures were heated at 100°C for 15 min and then quickly cooled in an ice bath. After centrifugation at 10,000 *g* for 20 min, the absorbance of the reaction solutions was recorded at 532 nm, 600 nm, and 450 nm. The MDA content of the reaction solutions was calculated as: MDA content (μmolL^-1^) = 6.45 (OD532–OD600)-0.56OD450, and final result of MDA was expressed as μmolg^-1^.

The super-oxide dismutase (SOD, EC 1.15.1.1) activity was measured by using nitro blue tetrazolium (NBT) method (Chen and Wang, 2006). After reaction, the color changed was measured at 560 nm, and one unit of SOD activity equaled the volume of extract needed to cause 50% inhibition of the color reaction and express as U g^-1^.

For peroxidase (POD, EC1.11.1.7) activity, enzyme extract (50 μl) was added to the reaction solution system containing 1 ml 0.3% H_2_O_2_, 0.95 ml 0.2% guaiacol, and 1 ml 50 mmol/L pH 7.0PBS, the absorbance change of the brown guaiacol at 470 nm was recorded for calculating POD activity. One POD unit of enzyme activity was defined as the absorbance increase because of guaiacol oxidation by 0.01 (Ug^-1^) (Chen and Wang, 2006).

The glutamine synthetase (GS, EC 6.3.1.2) activity was measured according Sun et al. (2009). The reaction system containing 50 mM imidazole, 18 mM ATP-Na_2_, 28 mM MgCl_2_, 25 mM hydroxylamine, 92 mM l-glutamate-Na, (pH 7.2). The enzyme reaction was terminated with 500 μl of stopping solution containing 370 mM FeCl3, 200 mM TCA, 700 mM HCl. After centrifugation (5 min, 13,000 *g*) 600 μl of supernatant was mixed with 300 μl of water, and read at 540 nm and expressed as ΔA. mg^-1^ protein h^-1^ and ΔA represents the change of absorption value.

The nitrate reductase (NR, EC 1.7.99.4) activity was determined by the method of Sun et al. (2009). The extract was incubated in a reaction mixture containing 100 mM potassium phosphate buffer (pH 7.4), 10 mM EDTA, 0.15 mM NADH, and 0.1 M KNO_3_ at 30°C for 30 min. The reaction was stopped by the addition of TCA. The absorbance of the supernatant was determined at 540 nm after adding sulfanilamide and *N*-(1-napthyl)-ethylenediamine-dihydrochloride. The units was expressed as μg g^-1^ FW h^-1^.

#### Morphological Characters

The rest of the seedlings, total 50 seedlings in each treatment (10 seedlings per pot and 5 pots per treatment) were harvested for the measurements of the root and shoot fresh and dry weight, seedling length, weight per unit height, and root shoot ratio. The root and shoot fresh weight of the plant from each pot was weighted by the electronic analytical balance (BSA224S, Sartorius, Taiwan) immediately after harvesting. After weighing of the fresh weight, the shoot part of the plant was used for measuring of the seedling length by a plastic ruler. Then the fresh sample of the root and shoot part of the plant was placed in the oven, dried at 80° C to constant weight for measurement of the root and shoot dry weight. The plant weight per unit seedling length and root/shoot ratio was determined with the following formulae:

Plant weight per unit seedling length (mg cm^-1^) = dry weight of the shoot/seedling length.

Root shoot weight ratio = dry weight of the root/dry weight of the shoot.

### Statistical Analysis

Analyses of variances (ANOVA) were performed by the Linear Model Procedure of Statistix version 8 (Statistix 8, Analystical, Tallahassee, FL, USA). Comparisons of means among different treatments were made according to the least significant difference (LSD) at the 5% probability level. The figures were made by using the SigmaPlot for windows version 10.0 (Systat Software Inc., San Jose, CA, USA).

## Results

### Analysis of Variance of the Investigated Parameters

All maize verities differed significantly regarding most of the investigated parameters in both sampling stags, except for Ci and NR activity in leaves at 3 DAT and for SOD activity and MDA content in root and GS and NR activity in leaves at 7 DAT. GABA application also affected some of the investigated parameters; nevertheless, no significant effect was noted for shoot weight per unit height, root shoot weight ratio, Ci, proline and MDA contents in leaves of both harvest stages. Moreover, activities of POD, GS, and NR in leaves at 3 DAT and root dry weight and GS activity in leaves at 7 DAT were also affected considerably. For variety and GABA interaction, the significant effects were observed for root and shoot fresh weight, Ci, SPAD values in leaves, protein content in both leaves and roots, SOD activity in leaves, and MDA content in roots at both 3 and 7 DAT (**Table 1**).

**Table 1:** Analysis of variance of the investigated parameters.

Index		3 DAT			7 DAT	
		
	V	T	V × T	V	T	V × T
Root fresh weight	16.56^∗∗^	76.52^∗∗^	6.41^∗^	47.10^∗∗^	17.47^∗∗^	4.51^∗^
Shoot fresh weight	34.16^∗∗^	34.25^∗∗^	6.77^∗^	14.87^∗∗^	44.48^∗∗^	4.40^∗^
Root dry weight	37.49^∗∗^	6.43^∗^	2.71ns	70.72^∗∗^	2.25ns	1.48ns
Shoot dry weight	25.47^∗∗^	8.62^∗^	2.44ns	25.38^∗∗^	43.28^∗∗^	1.41ns
Plant height	113.95^∗∗^	104.19^∗∗^	28.10^∗∗^	28.40^∗∗^	41.68^∗∗^	3.91ns
Shoot weight per unit height	18.70^∗∗^	0.01ns	0.78ns	41.20^∗∗^	0.40ns	2.19ns
Root shoot weight ratio	6.93^∗^	0.25ns	0.22ns	85.04^∗∗^	3.51ns	0.16ns
Pn in leaves	21.26^∗∗^	77.40^∗∗^	27.72^∗∗^	164.31^∗∗^	55.52^∗∗^	0.02ns
Gs in leaves	36.63^∗∗^	11.37^∗∗^	0.24ns	117.92^∗∗^	5.67^∗^	2.56ns
Ci in leaves	4.22ns	2.91ns	11.07^∗∗^	420.51^∗∗^	0.15ns	4.47^∗^
Tr in leaves	91.51^∗∗^	11.02^∗∗^	0.81ns	121.35^∗∗^	24.08^∗∗^	11.25^∗∗^
SPAD value in leaves	395.46^∗∗^	1.45ns	19.23^∗∗^	321.23^∗∗^	53.95^∗∗^	30.97^∗∗^
Protein content in leaves	233.57^∗∗^	50.01^∗∗^	43.22^∗∗^	117.87^∗∗^	105.90^∗∗^	80.67^∗∗^
Protein content in roots	7872.24^∗∗^	22971.2^∗∗^	7814.13^∗∗^	517.23^∗∗^	2437.08^∗∗^	628.13^∗∗^
Proline content in leaves	177.42^∗∗^	0.36ns	0.69ns	18.89^∗∗^	2.51ns	5.33^∗^
SOD activity in leaves	156.99^∗∗^	5.43^∗^	4.33^∗^	77.99^∗∗^	40.17^∗∗^	42.42^∗∗^
SOD activity in roots	59.71^∗∗^	35.88^∗∗^	23.41^∗∗^	1.47ns	8.96^∗^	0.97ns
POD activity in leaves	15.48^∗∗^	1.37ns	4.24ns	619.31^∗∗^	58.93^∗∗^	56.43^∗∗^
POD activity in roots	33.48^∗∗^	25.07^∗∗^	5.85^∗^	13.20^∗∗^	9.21^∗^	0.97ns
MDA content in leaves	32.50^∗∗^	0.00ns	0.46ns	56.00^∗∗^	0.06ns	0.23ns
MDA content in roots	300.57^∗∗^	70.40^∗∗^	12.43^∗∗^	0.07ns	9.51^∗^	8.71^∗∗^
GS activity in leaves	5.33^∗^	0.25ns	1.38ns	4.31ns	0.91ns	1.32ns
NR activity in leaves	4.31ns	0.91ns	1.32ns	4.62ns	12.28^∗∗^	2.56ns


*ns, non-significant at *P* < 0.05 level; ^∗^*P* < 0.05; ^∗∗^*P* < 0.01 levels.*

### Morphological Characters

**FIGURE 1:**
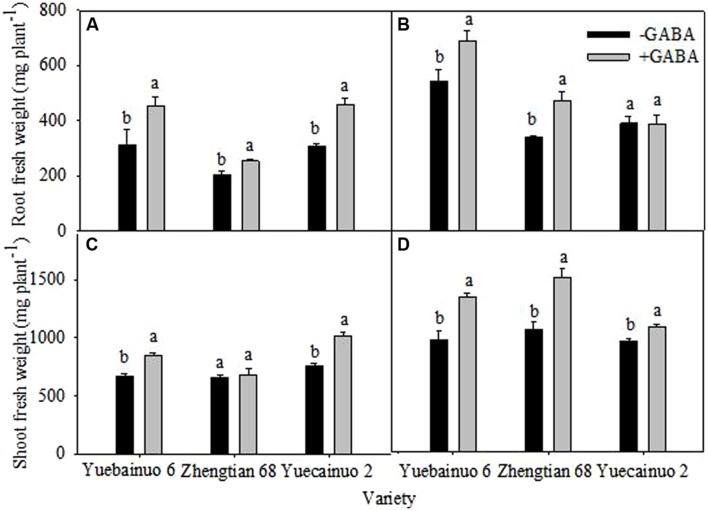
**Fresh weight of root and shoot of maize seedling at 3 DAT **(A,C)** and 7 DAT **(B,D)**.** Vertical bars with different lower case letters above are significantly different at *P* < 0.05 by LSD tests. Capped bars represent SD (*n* = 4).

γ-aminobutyric acid treated maize seedlings improved root fresh weight of Yuebainuo 6, Zhengtian 68, and Yuecainuo 2 at 3 DAT by 45.30, 23.57, and 49.45%, respectively, compared with non-treated seedlings (**Figure 1A**). At 7 DAT, GABA treated seedlings showed significant increase in root fresh weight by 26.82% for Yuebainuo 6 and by 38.88% for Zhengtian 68, but no significant difference was observed for Yuecainuo 2 (**Figure 1B**). With GABA application, the shoot fresh weight was improved up to 27.33, 3.52, and 33.09% for Yuebainuo 6, Zhengtian 68, and Yuecainuo 2, respectively, at 3 DAT, and the significance was detected for Yuebainuo 6 and Zhengtian 68 only whereas a remarkable increase in shoot fresh weight was observed for Yuebainuo 6, Zhengtian 68, and Yuecainuo 2 by 38.11, 42.33, and12.71%, respectively, at 7 DAT (**Figures 1C,D**).

**FIGURE 2:**
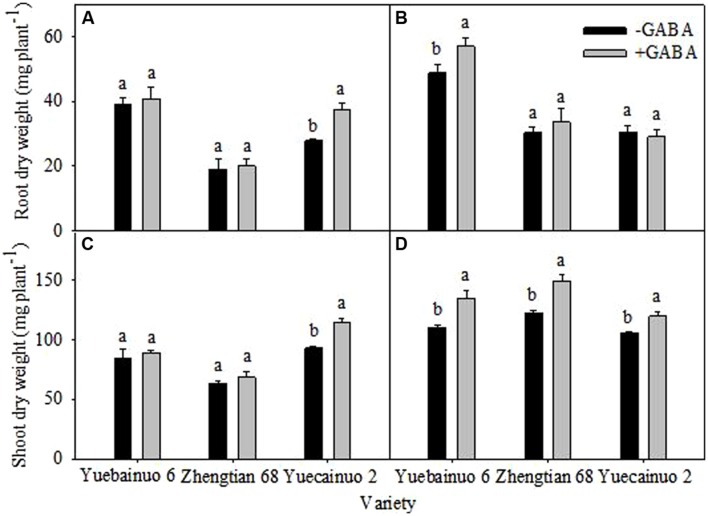
**Dry weight of root and shoot of maize seedling at 3 DAT **(A,C)** and 7 DAT **(B,D)**.** Vertical bars with different lower case letters above are significantly different at *P* < 0.05 by LSD tests. Capped bars represent SD (*n* = 4).

γ-aminobutyric acid treated seedlings improved root dry weight by 34.80% for Yuecainuo 2 at 3 DAT and by 16.60% for Yuebainuo 6 at 7 DAT (**Figures 2A,B**) whilst a notable increase in shoot was observed for Yuecainuo 2 (23.47%) at 3 DAT. Furthermore, a significant improvement in shoot dry weight was recorded for Yuebainuo 6, Zhengtian 68, and Yuecainuo 2 at 7 DAT by 22.10, 21.73, and 13.16%, respectively, (**Figures 2C,D**).

**FIGURE 3:**
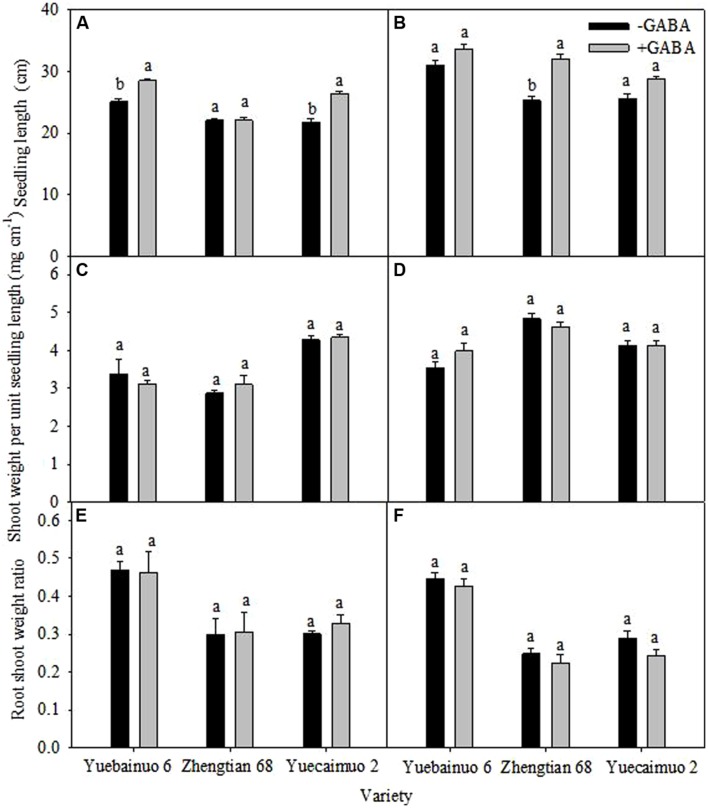
**Plant height, shoot weight per unit height, root shoot weight ratio of maize seedling at 3 DAT **(A,C,E)** and 7 DAT **(B,D,F)**.** Vertical bars with different lower case letters above are significantly different at *P* < 0.05 by LSD tests. Capped bars represent SD (*n* = 4).

Additionally, under GABA treatment, the seedling length was significantly improved for Yuebainuo 6 and Yuecainuo 2 at 3 DAT, and for Zhengtian 68 at 7 DAT, nevertheless, non-significant effect of GABA application was observed for the shoot weight per unit seedling length and root shoot weight ratio (**Figure 3**).

### Photosynthesis and Gas Exchange

**FIGURE 4:**
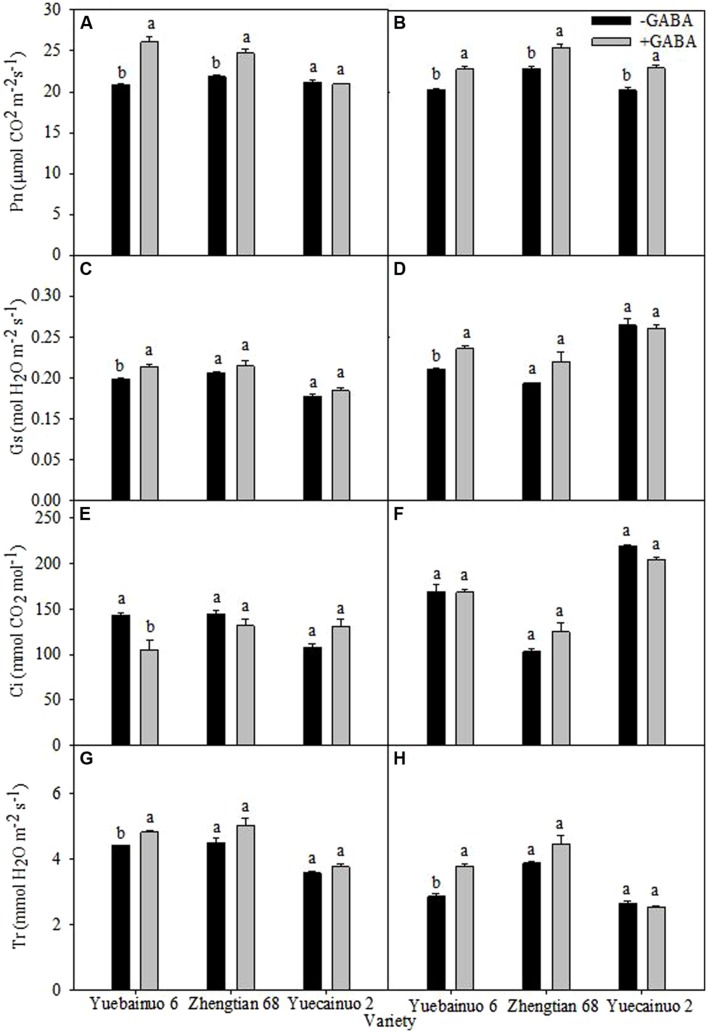
**Photosynthetic physiology of maize seedling at 3 DAT **(A,C,E,G)** and 7 DAT **(B,D,F,H)**.** Vertical bars with different lower case letters above are significantly different at *P* < 0.05 by LSD tests. Capped bars represent SD (*n* = 4).

γ-aminobutyric acid-induced enhancement in net photosynthetic rate (Pn) were recorded for Yuebainuo 6 and Zhengtian 68 with an increment of 25.13 and 12.94% at 3 DAT, respectively; however for Yuebainuo 6, Zhengtian 68, and Yuecainuo 2, the net photosynthesis rate were 12.45, 11.23, and 13.19% higher at 7 DAT, respectively. For stomatal conductance (Gs), significant increase was found for Yuebainuo 6 upto 9.75 and 15.91% at 3 and 7 DAT, respectively (**Figures 4C,D**), however, there was no significant effect of GABA on intercellular CO_2_ concentration (Ci) for all maize verities, except for a significant reduction of for Yuebainuo 6 at 3 DAT (**Figures 4E,F**). Interestingly, the transpiration rate (Tr) and stomatal conductance (Gs) showed similar trends for all three maize verities with an increase of 8.93 and 32.72% for Yuecainuo 6 at 3 and 7 DAT, respectively (**Figures 4G,H**).

### SPAD Value and Protein Content

**FIGURE 5:**
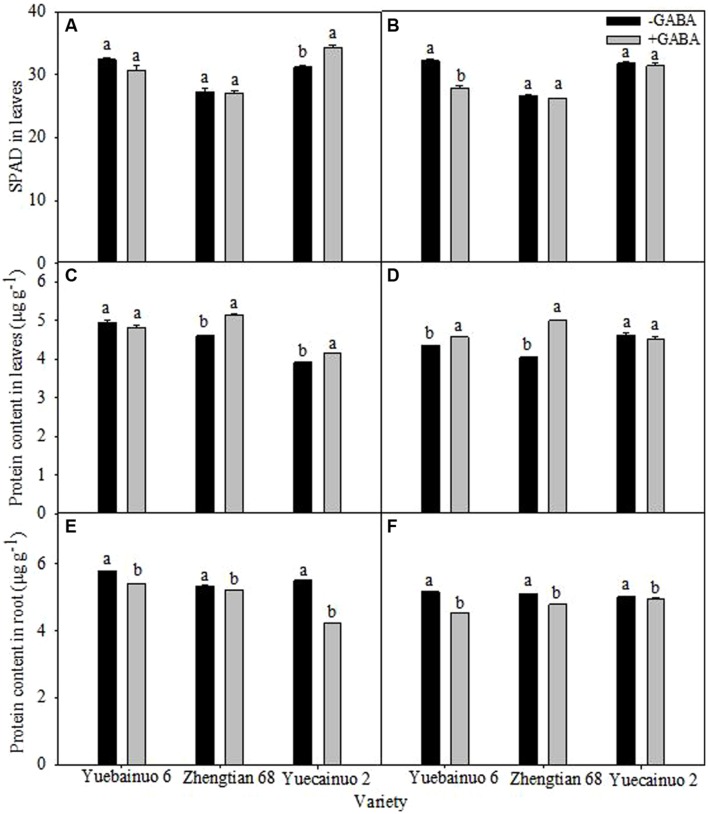
**SPAD value in leaves and protein content in leaves and root of maize seedling at 3 DAT **(A,C,E)** and 7 DAT **(B,D,F)**.** Vertical bars with different lower case letters above are significantly different at *P* < 0.05 by LSD tests. Capped bars represent SD (*n* = 4).

There was no significant effect of GABA on SPAD value in leaves was observed, besides the significant reduction of SPAD for Yuebainuo 6 at 7 DAT (**Figures 5A,B**). For protein content in leaves, GABA application significantly improved protein content of Zhengtian 68 and Yuecainuo 2 by 11.98 and 5.77%, respectively, at 3 DAT as well as significantly improved protein content of Yuebainuo 6 and Zhengtian 68 by 5.07 and 23.07%, respectively, at 7 DAT (**Figures 5C,D**). However, for protein content in root, significant reduction was recorded for all the varieties and both sampling stages (**Figures 5E,F**).

### Anti-Oxidant Enzymatic Activities

**FIGURE 6:**
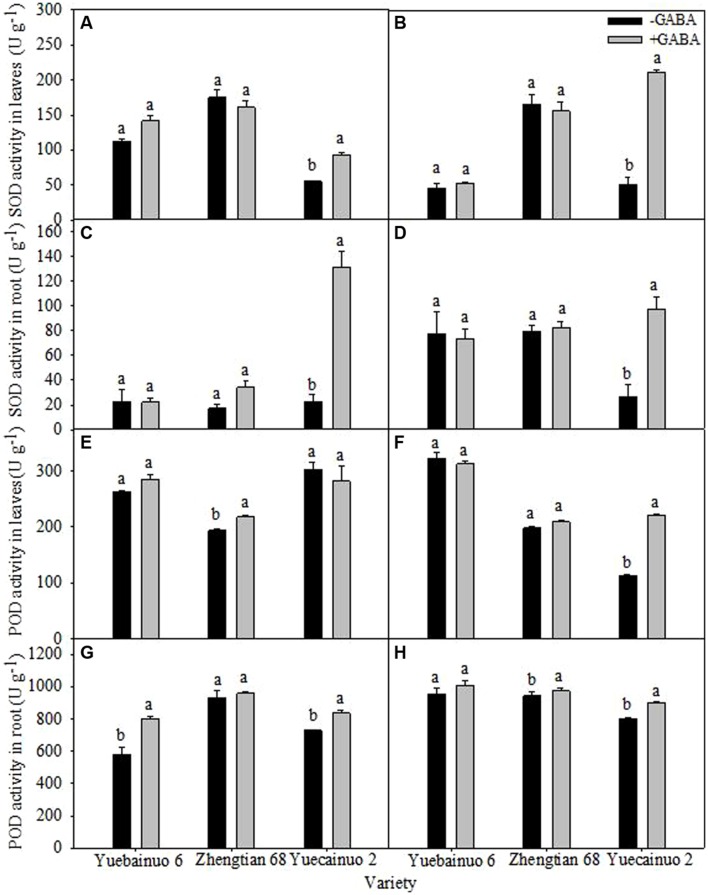
**Super-oxide dismutase and POD activity in leaves and root of maize seedling at 3 DAT **(A,C,E,G)** and 7 DAT **(B,D,F,H)**.** Vertical bars with different lower case letters above are significantly different at *P* < 0.05 by LSD tests. Capped bars represent SD (*n* = 4).

γ-aminobutyric acid treated maize seedling increased SOD activity in root and leaves of Yuecainuo 2 significantly, the increment in leaves was 68.91 and 307.53% at 3 and 7 DAT, respectively, and in roots was 484.21 and 262.16% at 3 and 7 DAT, respectively, (**Figures 6A–D**). Significant increment of POD activity in leaves was recorded for Zhengtian 68 (12.68%) at 3 DAT and for Yuecainuo 2 (95.30%) at 7 DAT (**Figures 6E,F**). For POD activity in root, the result showed 39.19, and 14.30%, higher POD activities in the roots of Yuebainuo 6 and Yuecainuo 2, respectively, at 3 DAT whereas for Zhengtian and Yuecainuo 2 root POD activities was 3.23 and 12.40% higher at 7 DAT than non-treated maize seedlings (**Figures 6G,H**).

### Proline Content and MDA Content

**FIGURE 7:**
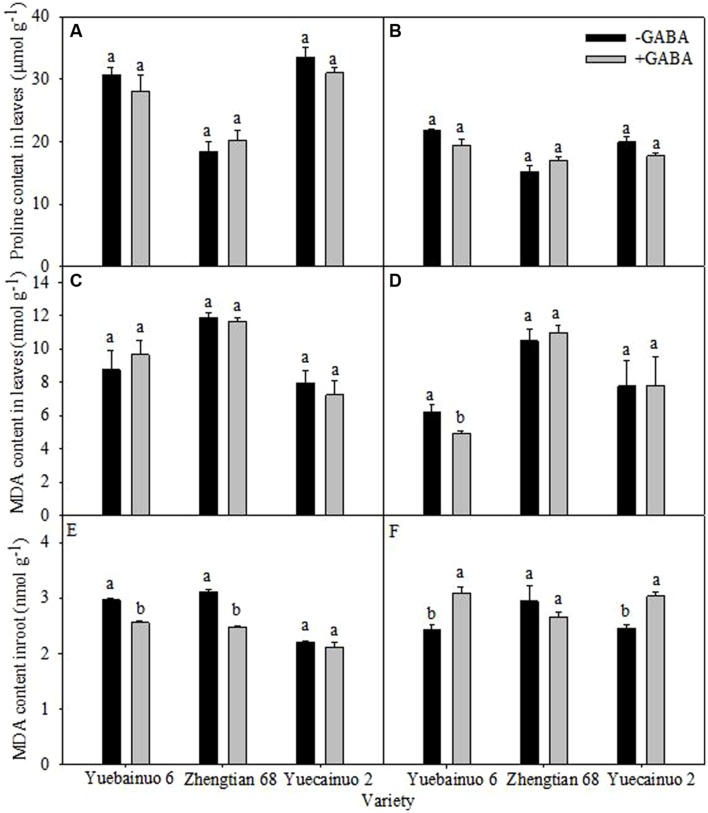
**Proline content in leaves and MDA in root and leaves of maize seedling at 3 DAT **(A,C,E)** and 7 DAT **(B,D,F)**.** Vertical bars with different lower case letters above are significantly different at *P* < 0.05 by LSD tests. Capped bars represent SD (*n* = 4).

Exogenous GABA application did not improved proline content in leaves of all maize verities significantly at both 3 and 7 DAT (**Figures 7A,B**). Moreover, at 3 DAT, GABA application reduced MDA contents in roots of Yuebainuo 6 and Zhengtian 68 significantly whereas in Yuecainuo 2 MDA contents were statistically similar with non-treated seedlings while all treated maize seedlings were remained statistically similar regarding leaves MDA contents. Furthermore, at 7 DAT, leaves MDA contents of Yuebainuo 6 were considerably lower than non-treated maize seedlings while Zhengtian 68 and Yuecainuo 2 similar leaves MDA contents as non-treated seedlings (-GABA). Interestingly, root MDA contents at 7 DAT were considerably higher in Yuebainuo 6 and Yuecainuo 2 under GABA treatment; nevertheless in Zhengtian 68 values for root MDA were marginally lower than non-treated maize seedlings but non-significant (**Figures 7C–F**).

### GS and NR Activity

**FIGURE 8:**
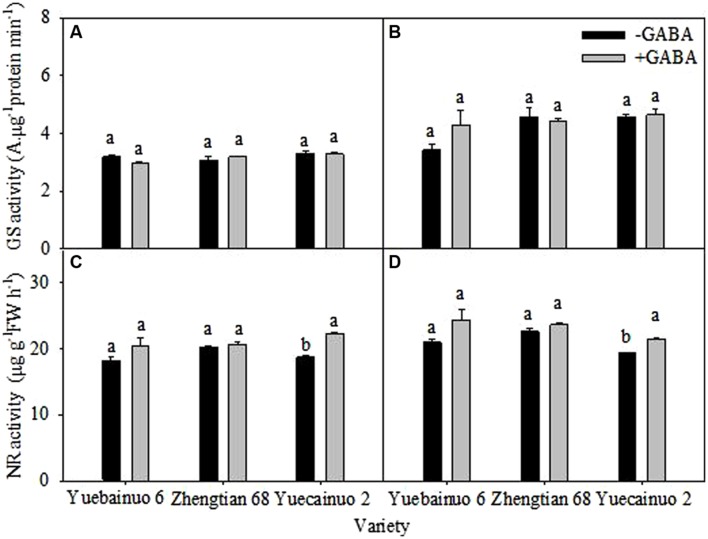
**Glutamine synthetase and NR activity in leaves of maize seedling at 3 DAT **(A,C)** and 7 DAT **(B,D)**.** Vertical bars with different lower case letters above are significantly different at *P* < 0.05 by LSD tests. Capped bars represent SD (*n* = 4).

There was no significant difference between with and without GABA treatments regarding GS activity for the three cultivars. For NR activity, the significant differences were observed for Yuecainuo 2 at both sampling stages, the increase in NR activity could be found for the other two cultivars; however, the values were marginally higher. Overall, the increment of NR activity in leaves were remained up to 12.06, 1.76, and 19.09% higher than –GABA at 3DAT for Yuecainuo 6, Zhengtian 68 and Yuecainuo2, respectively, whilst at 7 DAT, up to 16.13, 4.43, and 11.32% higher NR activities were observed in Yuecainuo 6, Zhengtian 68, and Yuecainuo 2, respectively (**Figure 8**)

### Correlation Analyses

**FIGURE 9:**
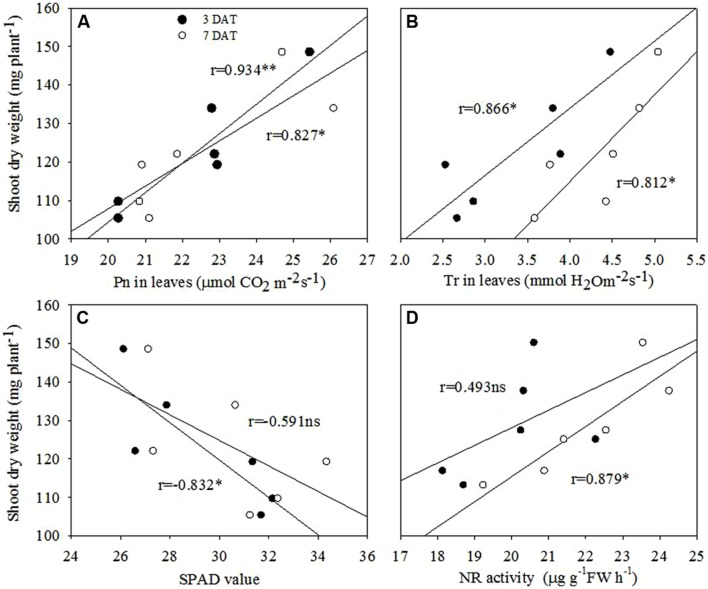
**Correlation analyses between **(A)** shoot dry weight and Pn in leaves, **(B)** shoot dry weight and Tr in leaves, **(C)** shoot dry weight and SPAD value, and **(D)** shoot dry weight and NR activity in leaves of maize seedling at 3 DAT and 7 DAT.** ns, non-significant at *P* < 0.05 level; ^∗^, significant at *P* < 0.05; ^∗∗^, significant at *P* < 0.01 levels.

Correlation analysis revealed that shoot dry weight at 7 DAT was in significantly and positively correlated with Pn and Tr in leaves at both 3 and 7 DAT. Positive associations were also recorded for the shoot dry weight at 7 DAT with NR activity in leaves, however, significance was recorded only for 7 DAT. Further, shoot dry weight at 7 DAT was negatively associated with SPAD values in leaves at both 3 and 7 DAT, but the significance was only recorded for 7 DAT (**Figure 9**).

## Discussion

γ-Aminobutyric acid (GABA), a non-protein amino acid, consists a considerable fractions of free amino acids in plant cells; however, its role in plants remained a bit unclear as compared to animal cells where its role as a neurotransmitter is well-recognized. Regulations in cytosolic pH, anti-oxidative enzymatic systems, buffering agent in C and N metabolism, osmoregulation, armoring against oxidative stress, and signal transduction are the major roles of GABA that might lead to improvement in overall plant performance (Kinnersley and Lin, 2000; Bouche and Fromm, 2004)

In present study, exogenous GABA application significantly improved morphological growth of seedlings of three maize cultivars, i.e., Yuecainuo 6, Zhengtian 68, and Yuecainuo 2 in terms of improved root-shoot fresh and dry biomass, seedling length, and root/shoot ratio (**Figures 1–3**). Previously, studies declared GABA-induced improvement in many plant species that might be due to improved photosynthetic activities, relative water contents, osmolyte accumulation, leaf turgor and other related physio-metabolical mechanisms (Deewatthanawong et al., 2010; Shang et al., 2011; Yang et al., 2011; Nayyar et al., 2014). GABA application might have promoted the maize seedlings growth by inciting cell elongation and division or/and by maintaining metabolic balance within plant tissues. Moreover, in another experiment, GABA application at 250 and 5 μM enhanced growth of *Stellaria longipes* and *Lemna*, respectively, under normal growing conditions (Kathiresan et al., 1998; Kinnersley and Lin, 2000).

Plant photosynthesis and transpiration rates are affected by various factors while improvements in photosynthetic yields and high photosynthetic acclimation would be of great interest in a crop like maize (Ashraf et al., 2016). Our results revealed that exogenous GABA application promoted net photosynthesis, gas exchange capacities and chlorophyll biosynthesis in all maize cultivars under study (**Figures 4** and **5**). GABA application related improvement in net photosynthesis and gas exchange in terms of stomatal conductance, intercellular CO_2_ concentration, transpiration rate in all maize cultivars of possibly due to maintenance of cell turgor, promoted chlorophyll biosynthesis, reduced oxidative damage by regulating various physio-biochemical processes. GABA could promote the synthesis of photosynthetic pigments and carotenoids that might be helpful in nurturing photosynthetic machinery. In a recent study, Vijayakumari and Puthur (2016) found a significant increase in the activities of photosystem I and II in *Piper nigrum* Linn. Plants, when seeds were primed with GABA. In contrast, effects of GABA application on net photosynthesis, chlorophyll contents and some parameters of chlorophyll fluorescence, i.e., maximal photochemical efficiency of photosystem II (Fv/Fm) and non-photochemical quenching coefficient (NPQ) were not improved significantly with GABA application whilst promotive effects were more prominent on electron transport rate (ETR), actual photochemical efficiency (ΦPSII) and photochemical quenching coefficient (qP) and Chl a/b in tomato seedlings grown in hydroponic culture (Luo et al., 2011). Strong shielding effects of GABA on photosynthetic machinery and chlorophyll contents in rice seedlings under heat stress were also reported by Nayyar et al. (2014).

Cytosolic accumulation of osmolytes is important to maintain cell turgor and involved in osmoregulation (Anjum et al., 2016). Our results indicated that GABA application enhanced protein contents in leaves of all maize cultivars while lowered in roots (**Figure 5**) whilst accumulation of proline concentration remained non-significant (**Figure 7**). Sustained photosynthesis, protection against protein and enzymes degradation as well as osmoregulation is the most apparent physiological effects of proline (Hare et al., 1999). Previously, GABA at 1, 5 10 mM was applied to peach fruit (*Prunus persica*) and its application at 5 mM led to significant increase in proline accumulation (Shang et al., 2011). Proline inducted protection of thylakoid membrane from oxidative damage caused by ROS is also of great importance (Delauney and Verma, 1993).

γ-aminobutyric acid induced regulations in enzymatic activities (GS and NR activity in leaves), lipid peroxidation (in terms of malnodialdehyde accumulation) and anti-oxidants (SOD and POD) in roots and leaves were represented in (**Figures 6–8**). Activities of GS and NR as well as anti-oxidants (SOD and POD) were found higher in seedlings supplemented with GABA (**Figure 6**), however, GABA application lowered MDA contents at 3 and 7 DAT except Yuebainuo 6 and Yuecainuo 2 at 7 DAT where values for MDA were significantly higher under GABA treated maize seedlings (**Figure 7**). GABA maintains C:N balance, nitrogen metabolism by regulating nitrogen continuing compounds (Kinnersley and Turano, 2000; Bouche and Fromm, 2004). During nitrogen destitution, its positive correlation was with nitrate influx during whole growth period of rape which evidences its involvement in nitrogen metabolism (Bown and Shelp, 1989; Beuve et al., 2004). GABA-induced regulations in nitrogen and carbon metabolism involved enzymes including NR, GS were also noted in *Arabidopsis thaliana* seedlings (Barbosa et al., 2010 whereas GABA-related modulation of GS activity in *Lemna minor*were also observed by Rhodes et al. (1975). Moreover, our results also demonstrated that GABA might have an effect on NR activity and phosphorylation (as activity of GR was increased due to GABA application), and might play important roles in *N*-metabolism.

On the other hand, generation of ROS often caused membrane damage and disintegration of various cellular structures and organelles thus cause ultimate cell death (Mittler et al., 2004; Ashraf et al., 2015; Anjum et al., 2016), however, timely action of SOD and POD against ROS in GABA treated maize seedlings to protect membrane damage indicated potential of GABA to in maintaining cell integrity. GABA-induced maintenance of higher activities of anti-oxidants is crucial to improve plants’ ability against oxidative stress. Our results corroborated with the outcomes of Shi et al. (2010) who resulted that GABA modified the activities of various anti-oxidants to scavenge ROS in *Caragana intermedia* plants. Furthermore, GABA-mediated shielding effect on membrane integrity by controlling lipid peroxidation was also observed by Song et al. (2010) in barley seedlings. Overall, GABA could improve the nitrogen metabolism, anti-oxidative defense and reduced lipid peroxidation in maize seedlings. However, in our experiment, we sprayed 50 mg/L GABA but no control was included with 50 mg/L of amonium or another amino acid. We can therefore not distinguish between specific GABA signaling effects from effects of nitrogen fertilization through the leaves or roots which suggest further research is needed in future. However, GABA could improve nitrogen metabolism and anti-oxidative defense in maize seedlings. Endogenous GABA concentrations can play a role as a signaling molecule at low concentrations (<10 μM) while at higher concentrations (>1 mM), its involvement in various physio-metabolical processes, C/N metabolism, osmoregulation, plant defense responses, cytosolic pH homeostasis and protection of plants from oxidative damage has great importance (Kinnersley and Lin, 2000; Bouche and Fromm, 2004).

## Conclusion

This study revealed that application of GABA could improve maize seedling growth; while the increment of morphological growth is associated with net photosynthetic rate and gas exchange capacities as well as the improved antioxidant enzyme activities to scavenge ROS. Nitrogen metabolism in terms of improved NR and GS activities were also noted under GABA treated maize seedlings. In future, further studies are still needed at molecular levels to get better insight of GABA involvement in physio-biochemical processes and nitrogen metabolism related to better performance of crop plants.

## Author Contributions

WL, JL, GL, and JH designed the research. WL, JL, GL, YL, LG, and WL performed the experiments and collected the data. UA, FH, WL, JH, and WL analyzed the data and wrote the manuscript. UA, FH, LG, YL, and XT edited the manuscript and provided guidance during experimentation.

## Conflict of Interest Statement

The authors declare that the research was conducted in the absence of any commercial or financial relationships that could be construed as a potential conflict of interest.
